# Brief Intervention as a Method to Reduce Z-Hypnotic Use by Older Adults: Feasibility Case Series

**DOI:** 10.2196/51862

**Published:** 2024-02-08

**Authors:** Maria Torheim Bjelkarøy, Tone Breines Simonsen, Tahreem Ghazal Siddiqui, Sigrid Halset, Socheat Cheng, Ramune Grambaite, Jūratė Šaltytė Benth, Jennifer Gerwing, Espen Saxhaug Kristoffersen, Christofer Lundqvist

**Affiliations:** 1 Institute of Clinical Medicine Faculty of Medicine University of Oslo, Campus Ahus Lørenskog Norway; 2 Health Services Research Unit Helsetjenesteforskning Akershus University Hosptial Lørenskog Norway; 3 Department of Geriatrics Akershus University Hospital Lørenskog Norway; 4 Department of Psychology Norwegian University of Science and Technology Trondheim Norway; 5 Department of Neurology Akershus University Hospital Lørenskog Norway; 6 Department of General Practice Institute of Health and Society University of Oslo Oslo Norway

**Keywords:** prescription medication misuse, older adults, brief intervention, z-drugs, benzodiazepine-related drugs, BZD-related drugs, z-hypnotic, intervention, feasibility, case series, insomnia, sleep, substance overuse, older adult, treatment, reduction, benzodiazepine, hypnotics

## Abstract

**Background:**

Z-hypnotics or z-drugs are commonly prescribed for insomnia and sleep difficulties in older adults. These drugs are associated with adverse events and dependence and are not recommended for long-term use. Despite evidence of older adults being more sensitive to a wide array of adverse events and clinical guidelines advocating limiting use, inappropriate use in this population is still prevalent. Previous intervention studies have focused mainly on prescriber information. Simple, individually focused intervention designs are less studied. Brief intervention (BI) is a simple, easily transferable method mainly used to treat patients at risk of alcohol overuse.

**Objective:**

Our objective was to design and test the feasibility and acceptability of a BI intervention adapted to address individual, inappropriate use of z-hypnotics among older adults. This preparatory study aimed to optimize the intervention in advance of a quantitative randomized controlled trial investigating the treatment effect in a larger population.

**Methods:**

This feasibility case series was conducted at Akershus University Hospital, Norway, in autumn 2021. We included 5 adults aged ≥65 years with long-term (≥4 weeks) use of z-hypnotics and 2 intervening physicians. Additionally, 2 study investigators contributed with process evaluation notes. The BI consists of information on the risk of inappropriate use and individualized advice on how to reduce use. The focus of the intervention is behavioral and aims, in cooperation with the patient and based on shared decision-making, to change patient behavior regarding sleep medication rather than physician-based detoxification and termination of z-hypnotic prescriptions. Qualitative and descriptive quantitative data were collected from intervening physicians, study investigators, and participants at baseline, immediately after the intervention, and at the 6-week follow-up.

**Results:**

Data were obtained from 2 physicians, 2 study investigators, and 5 participants (4 women) with a median age of 84 years. The average time spent on the BI consultation was 15 minutes. All 5 participants completed the intervention without problems. The participants and 2 intervening physicians reported the intervention as acceptable and were satisfied with the delivery of the intervention. After the intervention, 2 participants stopped their use of z-hypnotics completely and participated in the follow-up interview. Study investigators identified logistical challenges regarding location and time requirements. Identified aspects that may improve the intervention and reduce dropouts included revising the intervention content, focusing on rebound insomnia, adding an information leaflet, and supporting the patient in the period between the intervention and follow-up. The notion that the intervention should best be located and conducted by the patient’s own general practitioner was supported by the participants.

**Conclusions:**

We identified important aspects to improve the designed intervention and found that the BI is feasible and acceptable for incorporation into a larger randomized trial investigating the treatment effect of BI for reducing z-hypnotic use by older adults.

**Trial Registration:**

ClinicalTrials.gov NCT03162081; http://tinyurl.com/rmzx6brn

## Introduction

Sleep difficulties and symptoms of insomnia are common, experienced by up to 50% of older adults [1]. Prevalence is higher in the older population than the younger population, and not staying asleep is the most common complaint, followed by initiating sleep and nonrestorative sleep [1-4]. Sleep difficulties in older adults are often associated with comorbid conditions including obstructive sleep apnea, cardiovascular disease, restless legs, nocturia, depression, and neurological conditions, as well as side effects from medication use [3,5].

Z-hypnotics or z-drugs (zolpidem, zopiclone, and zaleplon) are commonly used in the treatment of insomnia and sleep difficulties in older adults. These drugs have been suggested to be milder and safer options than benzodiazepines with a shorter half-life and selective gamma-aminobutyric acid (GABA)–binding qualities [6]. They are now the dominant prescribed sleeping medication in many countries [7,8]. Even so, these drugs are associated with adverse events and dependence [9,10]. A meta-analysis concluded that 13 patients need to be treated for 1 patient to experience improved sleep, while the number needed to harm (any adverse event) is 6 patients [11]. Z-hypnotics are not recommended for more than 2 weeks to 4 weeks of continuous use [12,13], as such use has been associated with reduced sleep quality, reduced cognitive function, and reduced psychomotor skills, including increased risk of falls [11,14]. In our work investigating hospitalized older Norwegian adults, we found a substantial proportion using z-hypnotics on a regular basis with a further considerable share having addictive behavior related to their use [15]. Reducing the use of these drugs by older adults is associated with improved muscle strength, better sleep quality, improved quality of life, and reduced daytime fatigue [16,17]. Despite the risks associated with z-hypnotics, reducing their use seems difficult for patients, with initial rebound insomnia as a common withdrawal symptom [9].

Evidence supports brief interventions (BIs) for reducing z-hypnotic and benzodiazepine use [18]. A BI is a specific structured intervention method that aims to facilitate and encourage a behavioral change in the patient. We developed a BI based on the framework suggested by Babor and Higgins-Biddle [19]. This framework has previously been used as an early intervention for patients at risk of substance abuse and is effective in treating patients at risk of alcohol overuse. Our research team has demonstrated that the BI method is effective for reducing pain medication use in patients with medication-overuse headache [20,21]. Based on this, we suggest that an adapted BI may also be beneficial for reducing the use of z-hypnotics. The BI scheme is a short one-time intervention based on individual behavioral adaptation and shared decision-making. It offers possibilities for the intervening physician to provide individualized support for the participant. The focus is to reduce the use of potentially addictive medications, in this case z-hypnotics, and not as an intervention to specifically treat insomnia. Cognitive behavioral therapy for insomnia is the recommended first-line treatment for insomnia [22] and includes a range of different components including sleep hygiene. With the risk of experiencing rebound insomnia as a side effect of reducing the use of z-hypnotics, the individualized BI scheme is open to provide advice on sleep hygiene, although this is not the main focus of the BI itself. Our aim was to investigate the logistics, feasibility, and acceptability of using the BI with older adults who have inappropriate use of z-hypnotics, in order to optimize the intervention itself in preparation for a later full-scale randomized controlled trial (RCT).

## Methods

This feasibility study investigated the logistics, feasibility, and acceptability of our BI design for reducing the use of z-hypnotics by older adults. The study was conducted at Akershus University Hospital, Norway, during September 2021 to November 2021.

### Main Outcomes

The main outcomes for this study were the feasibility and acceptability of the intervention. Acceptability was tested based on indicated parameters [23-26] with an emphasis on the following: logistics including the costs and practicalities, burden and the perceived effort for participation, affective attitude and self-efficacy toward the intervention, and intervention coherence and perceived effectiveness. This was investigated using the following research questions:

- How do the logistics work out from the participating patient point of view?
- How do the logistics and administration work out from an organizing point of view?
- Are the instruments for data collection acceptable?
- Is the BI framework acceptable to perform from a physician point of view?
- Is the BI understandable and acceptable for the patients?
- How do the patients experience the attempt to reduce use of z-hypnotics?

The collected data consisted of qualitative measures and descriptive data collected at baseline and a 6-week follow-up.

### Participants

**Figure 1:**
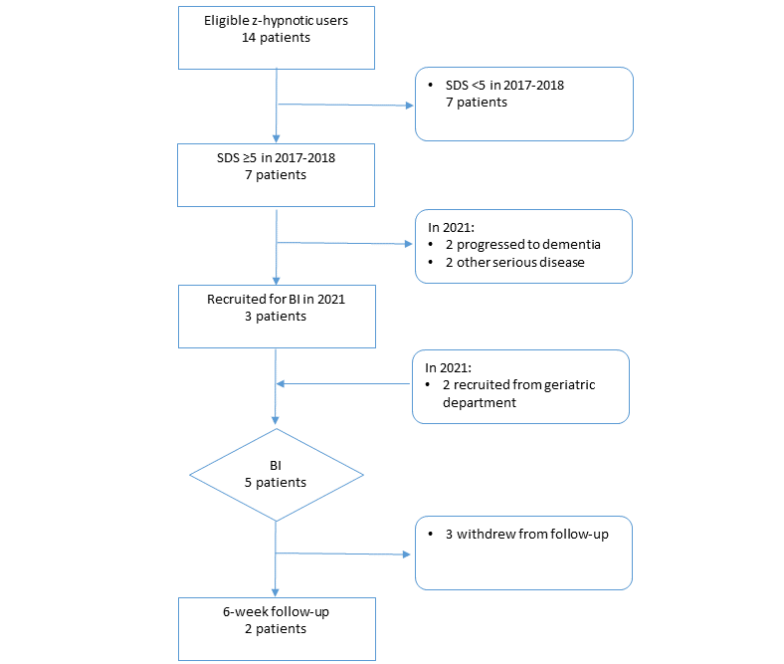
Study population flowchart. BI: brief intervention; SDS: Severity of Dependence Scale.

Adults aged 65 years and older with long-term (≥4 weeks) use of z-hypnotics were invited to participate. Prescription of z-hypnotics in Norway is exclusively approved for the purpose of sleep difficulties, with or without fulfilling the diagnostic criteria for insomnia [27,28]. Participants were recruited from eligible z-hypnotic users in an in-hospital observational study of 246 older adults originally conducted in 2017 and 2018 [15] as well as directly through the geriatric department at the hospital. The participation flowchart is presented in Figure 1.

**Figure 2:**
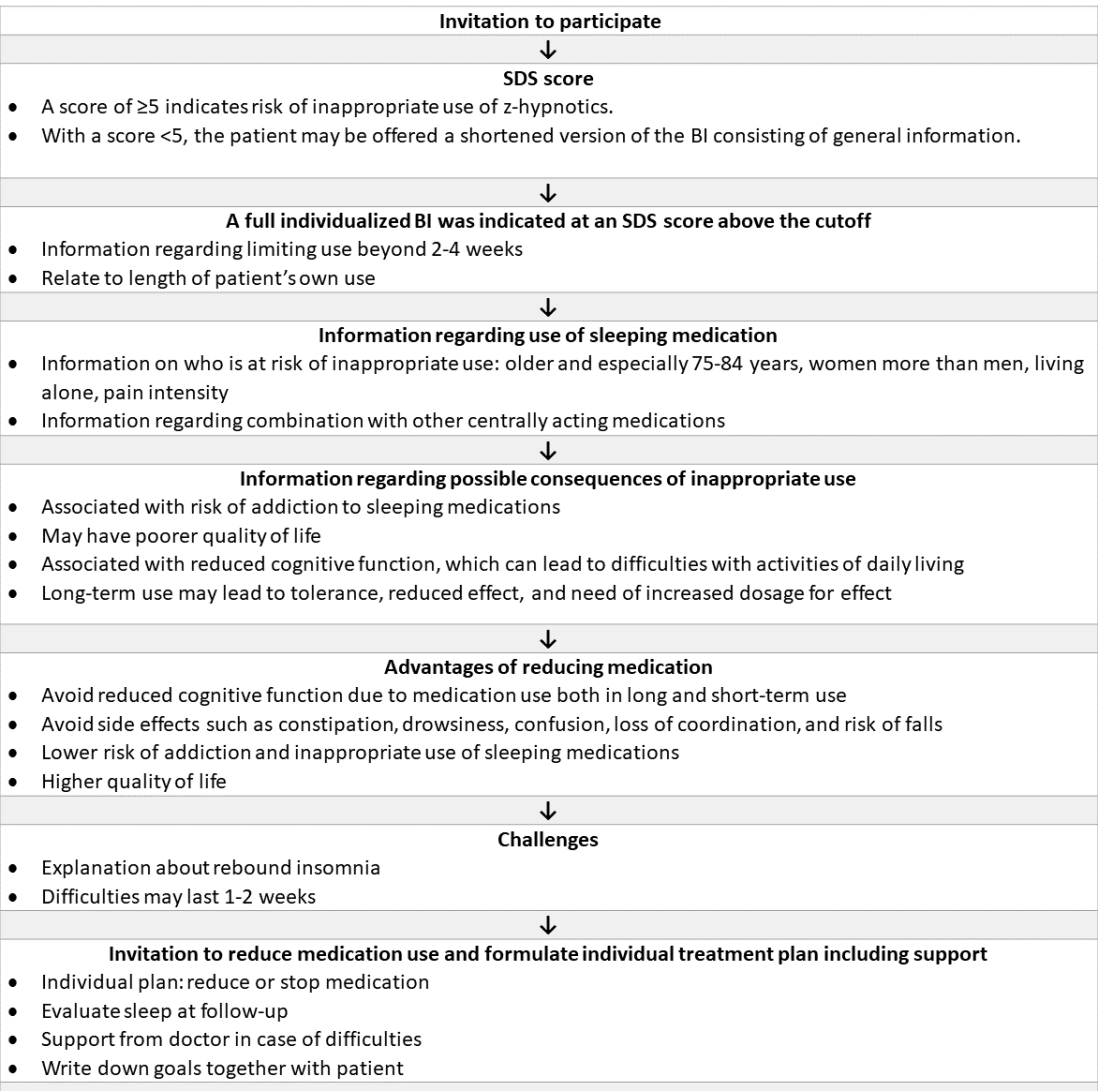
Brief intervention (BI) procedure. SDS: Severity of Dependence Scale.

We included 5 participants aged ≥65 years with previously reported inappropriate use of z-hypnotics. Inappropriate use was defined as using z-hypnotics for ≥5 days per week for ≥4 weeks based on clinical guidelines [12,13]. A score ≥5 on the 15-point Severity of Dependence Scale (SDS) indicated a risk of dependence [29]. Participants were not excluded if there was a discrepancy between previously reported inappropriate use and their current self-reported use during the BI intervention. As part of the individualized intervention, self-reported use as well as the SDS score were incorporated in the discussion as basis for the individual plan (see the description of the BI in Figure 2). Exclusion criteria consisted of having a Mini-Mental State Examination (MMSE) score ≤21, a serious visual or hearing impairment, insufficient Norwegian language skills, and the following pre-existing diagnoses: moderate to severe depression, stroke, dementia, or psychiatric disorders.

### Study Logistics

The intervention consisted of a baseline consultation and follow-up 6 weeks later. The baseline data collection was conducted in a hospital setting either in a consultation room or at the bedside by the study investigators (MTB and TBS). Subsequently, the BI consultation was conducted separately by 1 of 2 participating physicians (CL and SH). After the intervention session, both physicians and participating patients completed a short, written questionnaire with open-text responses regarding their experiences with the BI. The follow-up 6 weeks later involved a home visit by the study investigators (MTB and TBS) and consisted of questionnaires and a qualitative semistructured interview. This study was conducted during the COVID-19 pandemic, and care was taken with regards to safety measures and infection control.

### Brief Intervention

The developed BI scheme was based on the BI framework as described by Babor and Higgins-Biddle [19]. Based on studies previously conducted by our research group, we adapted the BI scheme for older adults using z-hypnotics [30,31].

Application of the BI was conducted in 2 steps. The first step consisted of providing training, information, and communication advice for the intervening physicians. The second step consisted of the physicians performing the BI scheme with the participants included in the study.

The BI conversation with the participant consisted of the following (full procedure outlined in Figure 2): identifying the risk of the participant’s dependency on z-hypnotics using the SDS questionnaire (Table 1), informing participants of their risk of inappropriate use and dependence, providing structured information using fact sheets about difficulties associated with reducing medication and possible withdrawal symptoms such as rebound insomnia [32,33], and adjusting individualized information to the participant’s own experience and inviting the participants to make a decision toward reducing or stopping use and to make a plan on how to proceed. The plan could include strategies on how to handle withdrawal symptoms including rebound insomnia and strategies for contact and support from a physician if needed.

**Table 1:** Questions in and scoring of the Severity of Dependence Scale (SDS).

Question number	Question content	Answer options and corresponding scores^a^
1	Do you think your use of sleeping pills is out of control?	Never/almost never=0, sometimes=1, often=2, always/nearly always=3
2	Does the prospect of missing a dose make you anxious or worried?	Never/almost never=0, sometimes=1, often=2, always/nearly always=3
3	Do you worry about your use of sleeping pills?	Never/almost never=0, sometimes=1, often=2, always/nearly always=3
4	Do you wish you could stop?	Never/almost never=0, sometimes=1, often=2, always/nearly always=3
5	How difficult do you find it to stop or go without your sleeping pills?	Not difficult=0, quite difficult=1, very difficult=2, impossible=3

^a^Each is scored on a 4-point scale (0-3), and the total maximum score is 15 points. In older adults using z-hypnotics, the cutoff score was ≥5 [26].

### Data Collection and Instruments

The main focus was to test the experience with, acceptability of, and logistics of the entire BI including the incorporation of quantitative assessment instruments that would be needed in a full-scale RCT. In this study, however, the demographic information and instruments were only used as descriptive information. The data collected included demographic information; the MMSE [34]; health-related quality of life measured with the EuroQol Group’s EQ-5D-3L [35]; the 6-item De Jong Gierveld Loneliness Scale [36]; the Hospital Anxiety and Depression Scale [37]; visual analogue scale (VAS) [38] scores for intensity of pain, anxiety, and depression; questions on experiences with pain; questions on experiences with sleep difficulties; the Bergen Insomnia Scale (BIS) [39]; the PROMIS-57 Profile v2.1 for sleep quality [40]; the clock test [41]; Cognistat [42]; the SDS [29]; the Cumulative Illness Rating Score-Geriatrics [43]; the Barthel Index for Activity of Daily Living [44]; the single leg balance test [45]; a record of medications and dosage; the degree of expectations and beliefs in the intervention and outcome measured using a VAS at baseline; and a written plan with individual goals for reducing z-hypnotics. A questionnaire on the experience delivering the BI was collected from physicians after each session. Process evaluation notes were obtained from study investigators.

At the 6-week follow-up, we also collected the experiences with the intervention and outcome using a VAS and a medication diary (z-hypnotics) for the past 6 weeks. We also conducted a semistructured qualitative interview (interview guide in Multimedia Appendix 1) evaluating the participants’ experiences with the BI conversation and their experiences during the 6-week follow-up period. The qualitative interview was tape-recorded and subsequently transcribed verbatim and analyzed.

### Analysis

The qualitative data were analyzed by investigators MTB and TBS using the text condensation method [46], and findings are reported as quotations. ELAN computer software (version 6.3; Max Planck Institute for Psycholinguistics) was used to transcribe the interviews. SPSS for Windows (version 26.0; IBM Corp) was used to record quantitative data. Quantitative data are reported descriptively.

### Ethical Considerations

This study was approved by the Regional Committee for Medical and Health Research Ethics (REK) (2016/2289) and the Akershus University Hospital data protection officer (PVO; 17-054). All participation was by written informed consent. Collected data were analyzed and stored de-identified as required by the REK and PVO. Data were stored on a protected server approved by the Akershus University Hospital PVO. Participants received no financial nor other compensation for their participation.

### Patient and Public Involvement

The Health Services Research Unit User Advisory Board at Akershus University Hospital reviewed and provided advice about the study. The board includes both patient representatives and representatives of the health services as well as other public representatives.

## Results

### Participants

We recruited 5 older adults (4 women) with a median age of 84 years and ≥4 weeks of z-hypnotic use. We recruited 3 of the 5 participants from eligible participants in our previous hospital-based observational study [15,29,47-50], and 2 participants were recruited directly through the geriatric department. The study population flowchart is presented in Figure 1.

Of the 5 participants, 4 reported using z-hypnotics ≥6 days per week, and 1 participant reported using z-hypnotics 1 day per week at baseline. The median number of days per week using z-hypnotics was 7 days. The median SDS score was 5. The median BIS score was 27 (max score 42), and 3 participants reported that they believed that they would have greater sleep disturbance. The main reasons for sleep disturbance included extensive daytime napping, nocturia, pain, and lying in bed thinking. Demographic and clinical characteristics of the participants are presented in Table 2. At baseline, 3 participants reported that their current experience with not regularly taking their z-hypnotics resulted in greater sleep disturbance, and 3 patients reported that they believed that they would have greater sleep disturbance if they did not take their sleeping medication (Table 2).

**Table 2:** Demographic data for the study population and beliefs about sleeping medication at baseline.

Characteristics		Participant 1	Participant 2	Participant 3	Participant 4	Participant 5
**Baseline**						
	Sex	Male	Female	Female	Female	Female
	Age (years)	81	70	84	87	89
	MMSE^a^ score	29	29	30	29	29
	BIS^b^ score	22	4	27	39	0
	Insomnia^c^	Yes	No	Yes	Yes	No
	SDS^d^ score	3	5	5	5	3
	Use of medication (days per week)	7	7	1	6	7
	Medication	Zopiclone	Zopiclone	Zolpidem	Zolpidem	Zopiclone
	EQ-5D VAS^e^ score	45	80	70	10	50
	Cognistat score	70	67	65	66	61
	Main reason for sleep disturbance	Daytime napping 4-5 times/day	Lay in bed thinking	Nocturia 3 times/night	Pain, unsettled, and thinking	Sleep itself and thinking
	CIRS-G^f^	8	9	8	10	11
**Current beliefs**						
	Sleep without meds^g^	Unsure if there is a difference	Worse	Worse	Worse	Do not know, always take it
	Belief about sleep without meds^h^	As good	Worse	Worse	Worse	Not sure

^a^MMSE: Mini-Mental State Examination.

^b^BIS: Bergen Insomnia Scale (range 0-42).

^c^Occurrence of insomnia according to the Bergen Insomnia Scale.

^d^SDS: Severity of Dependence Scale.

^e^VAS: visual analogue scale.

^f^CIRS-G: Cumulative Illness Rating Score-Geriatrics.

^g^“How have you slept the nights you have not taken sleeping pills?”

^h^“How do you think you will sleep if you do not take your sleeping pills?”

### Intervention Logistics, Physicians’ Experience, and Feasibility

The median duration of the BI consultation was 15 minutes. The 2 intervening physicians reported that the BI framework for a potentially sensitive subject made the doctor-patient consultation easier to conduct (Textbox 1, quotes 1 and 2). They also reported that performing the BI in a consultation room was preferred over a bedside setting. They further reported that all 5 participants were open and positive toward the BI conversation (Textbox 1, quotes 2 and 3), as demonstrated by the participants listening actively and asking questions. The tools for patient communication that were received during training for the BI were beneficial during the consultation. The most beneficial tools for communication included asking open questions, letting the participants talk about their own experiences, having the participant repeat information, and writing down information together with the participant. The study investigators identified that the number of instruments used and the travel to participants were time consuming and advised making adjustments toward limiting time consumption.

**Individual quotations from physicians immediately after the brief intervention (BI) consultation and individual quotations from the participating patients at the 6-week follow-up.**

**Quotations from the intervening physicians immediately after the BI consultation:**

1. “Overall positive atmosphere. Patient understood the content and stated that effect on cognitive function made an impression. Patient stated little belief in health advantages of changing medication use and stated that, at her age, it did not matter.”

2. “The BI was overall received with a positive attitude. Patient stated that she found quitting difficult as she was living alone and was afraid of not falling asleep.”

3. “Patient was open-minded, interested, and motivated to try to change medication use. Was aware of possibilities of medication misuse in general.”

**Quotations from the participants at the 6-week follow-up:**

4. “I stopped the sleeping pills that same night and have not touched them since. [...] I thought, that pill I will manage without, and that has gone very well. And after that, I have realized that I do not need to take them. [...] It does not take me any longer to fall asleep, and when I sleep, I sleep well. [...] It has been a relief not having to remember to take that pill.”

5. “Trying to stop the sleeping medication has been very hard. [...] I have not slept more than half of what I should have. Normally, I am up for the toilet 1 to 2 times a night, but throughout this period, I have been up 3 to 5 times each night, and that leads to poor sleeping. [...] So, therefore, I had decided to tell you that I cannot have it like this in the future. I am so old, maybe I will live 2 more years. It does not matter. I rather have good nights than to become a hundred years.”

6. “It [the BI] was a mild form of advice. I believe in that type of approach. Doctors have tried to scare me about things before, for example, when they wanted me to quit smoking.”

7. “I experience that what they said at the hospital [the BI] and what my general practitioner has said earlier; that information corresponds. That is good for me to hear.”

### Participants’ Experiences and Feasibility at Baseline

All 5 participants attending the BI consultation were positive overall toward participating and interested in the BI conversation. Expectations and beliefs measured by the VAS immediately after the intervention found median expectations of reducing medication use of 35 (min;max 3;100), median beliefs about improved health with reducing z-hypnotic use of 33 (min;max 2;100), median beliefs about the importance of reducing the medication of 27 (min;max 2;100), and beliefs in one’s own ability to reduce z-hypnotic use of 42 (min;max 1;100). Expectations and beliefs are presented in Table 3.

The participants’ intentions and goals on how to proceed after the BI were recorded, and 2 participants decided to quit z-hypnotics completely and immediately. Of the remaining 3 participants, 1 wanted to reduce dosage but continue regular use, 1 wanted to discuss a change to the type of z-hypnotic used with her general practitioner (GP), and 1 aimed to continue use as before and reported that this was about once a month.

The greatest challenge for reducing or stopping z-hypnotic use was reported by 3 participants as a worry that they would not sleep. One stated that she was not sure if changing medication mattered, as she was old and was soon going to die. One participant reported a need for support from the physician to stay motivated to stop z-hypnotic use, and 4 participants did not set up a direct plan for what they would do if they encountered difficulties.

**Table 3:** Expectations and motivation at baseline versus experiences at follow-up.

Measurements		Participant 1	Participant 2	Participant 3	Participant 4	Participant 5
Sex		Male	Female	Female	Female	Female
**Baseline expectations and motivation (VAS^a^ score)**						
	Expectations for reduction^b^	100	20	3	54	35
	Belief in health improvement^c^	100	33	2	84	27
	Importance to reduce meds^d^	100	27	2	23	10
	Belief in ability to reduce meds^e^	100	53	1	42	10
**Experience at the follow-up (VAS score)**						
	Satisfied with the experience^f^	100	—^g^	—	15	—
	Health improvement^h^	1	—	—	7	—
	Experienced importance^i^	83	—	—	93	—
	Ability to adjust meds^j^	100	—	—	89	—

^a^VAS: visual analogue scale.

^b^”What are your expectations for trying to reduce the use of sleeping pills?” (VAS: 0=no expectations, 100=great expectations).

^c^“How much do you believe you will experience a health improvement if you reduce the use of sleeping pills?” (VAS: 0=no belief, 100=great belief).

^d^“How important is it for you to reduce or stop using the sleeping pill after talking to the doctor?” (VAS: 0=not important, 100=very important).

^e^“How sure are you that you can reduce or stop using sleeping pills if you decide to do so?” (VAS: 0=not sure, 100=very sure).

^f^“How satisfied are you with the experience of trying to reduce the use of sleeping pills?” (VAS: 0=not satisfied, 100=very satisfied).

^g^Not answered by this participant.

^h^“Did you experience a health improvement by reducing the use of sleeping pills?” (VAS: 0=no improvement, 100=great improvement).

^i^“How important was it for you to reduce or stop using the sleeping pills after talking to the doctor?” (VAS: 0=not important, 100=very important).

^j^“To what extent were you able to adjust the consumption of sleeping pills in relation to what you decided after consultation with the doctor?” (VAS: 0=no degree, 100=great degree).

### Logistics and Participants’ Experiences With Reducing Z-Hypnotics

At the 6-week follow-up, 2 participants completed the questionnaire and qualitative interview. Both managed to completely stop their use of z-hypnotics. Although 1 participant reported that stopping was easy and did not notice any difference (Textbox 1, quote 4), the other participant reported that trying to reduce medication had been very hard and aimed to start the medication again in the hope of getting more sleep (Textbox 1, quote 5). Of the 3 participants who decided to not participate in the follow-up interview, 1 stated a preference to engage in conversation about these types of medications with her GP, 1 reported having too much going on at home with disease in the family, and 1 participant was lost to follow-up. Experiences at the 6-week follow-up are presented in Table 3.

### Participant Feedback During Interviews at the 6-Week Follow-Up

Regarding the practicalities of participating, the 2 participants stated that it had been very easy, with no burden regarding cost or time requirements. They added that there would have been difficulties with participating at the follow-up if it had not been arranged as a home visit, as travelling to the hospital was difficult. Both stated that the BI conversation itself had been a good experience and that the information conveyed had been understandable (Textbox 1, quotes 6 and 7). They had both been aware of the negative side effects of z-hypnotics before the BI conversation and stated that they had not learned anything new in the BI conversation. At the follow-up, they could not recall any specific piece of information that had stood out regarding risks for long-term use, possible adverse events, or advantage for reducing use.

## Discussion

### Principal Findings

In this study investigating the feasibility of a BI for older adults with inappropriate use of z-hypnotics, we identified important aspects to improve before we proceed with the intervention. The focus for this study was to test the method, logistics, and intervention coherence, identifying strengths and weaknesses to improve the protocol for a larger study investigating the treatment effect. We tested the logistics and found that it is advantageous for the intervention to be delivered in primary care as opposed to secondary care; we need to reduce the number of data collection instruments; the designed BI communication framework was acceptable overall for both physicians and participants; and the participants found it challenging to participate further and reduce their medication, which in turn calls for further support during the intervention period.

Previous research has concluded that long-term use of z-hypnotics by older adults is to be avoided [12,13] and that the treatment effect of z-hypnotics is questionable [8]. It is suggested that attention should be directed at educational interventions [8], and such interventions have been found beneficial [51-53]. The BI method has been proven effective at handling substance overuse and dependence [19,20]. Adjusting the BI to benefit older adults with inappropriate use of z-hypnotics is probably appropriate and valuable for both individual patients and health care professionals and systems.

### Evaluation of Study Logistics

Female gender and being ≥75 years of age are associated with a greater risk of inappropriate use [15], hence we believe we tested our intervention in a relevant population. Recruiting and intervention were linked to a previous observational study [15] and conducted in a hospital setting, which we decided was the most cost-effective and practical solution to test both the study logistics and intervention under the current circumstances. The hospital setting is a good opportunity for revising patients’ medications according to START/STOP criteria [54]. Conducting the intervention in a specialist environment at the secondary health care level was theorized to carry some impact for adherence. We did, however, experience that this type of intervention may be better suited to the primary care setting where it can occur as a conversation between patients and their GPs. Prescriptions for z-hypnotics, although often initiated during a hospital stay, are commonly continued by the GP. Training GPs to perform the BI may increase awareness of prescription habits regarding z-hypnotics. One study participant reported her preference for discussing issues regarding her use of z-hypnotics directly with her GP as the reason for withdrawing from the follow-up interview. Although the participants reported no burden and low perceived effort with participating in the study, they noted not having to travel to the hospital was important for participation. GPs’ proximity to their patients’ locale and their everyday involvement in their patients’ health issues may provide a more effective and suitable setting for this intervention.

The average duration of the BI was 15 minutes, which is longer than anticipated. During the design, we aimed for a 10-minute intervention, as we have previously experienced this duration to be acceptable [20,30]. We believe that limiting time spent on the intervention could increase feasibility in a busy daily practice for both patients and GPs, and it may be advantageous to revise the BI scheme accordingly. However, the increase in expected duration could be manageable for GPs during a conversation with older patients.

Both at baseline and follow-up, the participants completed a series of questionnaires, patient-reported outcomes forms, and tests. As these were in addition to the BI itself, participating in the study was both time-consuming and demanding. In a future study investigating the treatment effect, limiting data collection would be advantageous for both reducing load and possibly increasing adherence at follow-up.

### Evaluation of Patient Intervention Coherence

Core to this study was investigating the participants’ own understanding of the intervention and their thoughts and beliefs regarding it. To get an understanding of how the intervention would affect the participants, we also investigated their thoughts and beliefs regarding sleep disturbance and z-hypnotics.

In our study, reported reasons for not sleeping included extensive daytime napping (poor sleep hygiene), nocturia, and pain. Once awake, lying in bed thinking was reported as the most common reason for not sleeping. When adjusting for comorbidities, pain, depression, and medication use, which are all factors associated with sleep disturbance, the prevalence of insomnia, as defined by the International Classification of Sleep Disorders (3rd edition) [27,28,55] criteria, decreases in older adults [3,56]. This calls into question the appropriateness of pharmacological treatment for sleep disturbance in older adults and underlines the importance of identifying all issues regarding sleep disturbance during diagnosis and treatment. Reasons for sleep disturbance may have an impact on the measured effect of an intervention study, indicating a benefit for adjusting for this in the study design.

When reporting on their pre-intervention experience with the use of z-hypnotics, the participants stated that, during nights when they had not taken their z-hypnotics, they would have a worse night. Further, they reported anticipating having a worse night if they did not take their z-hypnotics. Rebound insomnia is a known symptom of z-hypnotic withdrawal [32]. It is most prevalent during the first days of withdrawal, and the patient may interpret this as confirmation that they cannot sleep without their medication. In addition, research has shown that there is a large placebo effect with z-hypnotics. The difference in sleep latency between active treatment and placebo is only 22 minutes of objectively measured effect in favor of active treatment [8], demonstrating a very low clinical gain of active treatment compared with placebo and underlining anticipation as a considerable portion of the experienced effect. Further, only 50% of patients are aware that long-term use of z-hypnotics can reduce sleep quality, and up to 84% of patients are aware of dependence, interaction with alcohol use, and dizziness. Regardless of the awareness of side effects, only 26% of patients report an interest in reducing their use of z-hypnotics [57]. Not being aware of possible side effects and placebo effects in addition to experiencing rebound insomnia with withdrawal are factors that will affect intervention compliance and patients’ interest in making changes to their z-hypnotics.

At the follow-up interviews, the participants reported that they could not recall information shared during the BI consultation regarding use of z-hypnotics, risk of adverse events, and benefits from reducing use. They stated that they did not learn anything new about z-hypnotics during the consultation but that the BI consultation had confirmed some previous knowledge. This underlines the importance of investigating the patients’ knowledge about the substance in question. Awareness about patients’ knowledge is important on an individual level. It emphasizes the importance of revising the *content* and *delivery* of the information about z-hypnotics presented in the BI. It also prompts exploring more aspects of the BI technique such as providing a short information leaflet [19] that summarizes and repeats the core information given at the consultation. In this study, we chose not to use such additions, as we wanted to explore the feasibility of using just a single consultation.

Another aspect of the BI technique we chose not to pursue in this study was to contact participants to offer support during the follow-up period. We regarded 6 weeks to be a relatively short follow-up period and therefore did not actively seek contact. As part of the BI, the participants were invited to make a plan about what to do in case they had challenges reducing their medication. They were given a phone number to contact if they had questions, needed support, or encountered difficulties. One participant reported needing support from the physicians during the follow-up, but 4 did not make a plan for what to do if they met with challenges. Prompting patients to create a plan about what do to if they meet with difficulties and contacting the patients during the weeks after the intervention may improve participation and adherence.

### Strengths and Weaknesses

This feasibility case series was performed as part of our development of a complex intervention [58], as suggested by the UK Medical Research Council, in which a central component is conducting a feasibility study [58,60]. It provided useful information regarding the feasibility, acceptability, and logistics. Naturally, such a small feasibility series does not contribute quantitative data nor solve the central question about whether the BI is an effective intervention. It was a valuable opportunity for an in-depth investigation of the BI scheme with older adults using z-hypnotics. It also provided information about older adults’ thoughts and beliefs about sleep disturbance and their medication use. The main weakness was the small sample as well as the attrition of participants: 3 of 5 participants declined the follow-up interview. We found, however, that both the participants who completed and those who withdrew provided valuable information to further design and optimize the BI scheme for older adults using z-hypnotics.

### Conclusion

This study assessing the feasibility of the BI design for older adults with inappropriate use of z-hypnotics identified some important aspects with regards to improving the design before proceeding to a larger study investigating the treatment effect. Conducting the BI in primary care, limiting the duration of the BI while emphasizing core information, providing patients with an information leaflet, and contacting patients for support may improve the effect of the intervention.

## Appendix

Multimedia Appendix 1 Interview guide.

## Abbreviations

BI: brief intervention
BIS: Bergen Insomnia Scale
GABA: gamma-aminobutyric acid
GP: general practitioner
MMSE: Mini-Mental State Examination
PVO: data protection officer
RCT: randomized controlled trial
REK: Regional Committee for Medical and Health Research Ethics
SDS: Severity of Dependence Scale
VAS: visual analogue scale

## Data Availability

The raw data are closed for public access due to data protection regulations by the Akershus University Hospital data protection officer and the Regional Committees for Medical and Health Research Ethics. However, summarized data may be available upon reasonable request to the corresponding author MTB.
