# Supplementary material for: Brief Intervention as a Method to Reduce Z-Hypnotic Use by Older Adults: Feasibility Case Series
Source: JMIR Form Res. 2024 Feb 8;8:e51862. doi: 10.2196/51862 (PMC10884901; doi:10.2196/51862)
Supplement: Multimedia Appendix 1 [file formative_v8i1e51862_app1.pdf]

Interview guide: Translated version in English
Patients' experience – follow-up week six

Introduction:

Last time we met, you had a conversation with the doctor about using sleeping pills. The doctor showed you some charts and you completed a test that showed that you had a low/somewhat/high risk of becoming addicted to your sleeping pills. Together with the doctor you decided to change/reduce/stop your medication. Together with the doctor you set some goals for the following weeks.

Interview:

1. How do you feel that this has been? (easy introduction)

2. Is there anything in particular that you can remember from the conversation you had with the doctor?

3. I would like to ask you about your thoughts on reducing sleeping pills. If you think back to that day; on a scale from 0 to 10, where 0 is no belief that you would be able to change your medication use and 10 is great belief; how much faith did you have in your own abilities to change use of sleeping pills after the conversation with the doctor?
    a. What do you think is the reason for it not being a (mention a lower number than patient has provided)?
    b. What do you think is needed for it to be (mention a higher number than the one the patient provided)?

4. Did you learn anything new or unexpected about sleeping pills during the conversation with the doctor?

5. After the meeting with the doctor you filled out a form in which you had to indicate your expectations for reducing use of sleeping pills. If you think back now, what were your expectations at the time regarding this?
    a. What do you think about those expectations now?

6. How demanding or challenging has it been for you to try to change your use of sleeping pills? On a scale from 0 to 10 where 10 is easy and no challenge and 10 is very demanding or challenging?
    a. Which challenges have you encountered?

    b. How have you solved it when you have encountered these challenges?
    c. Looking back now, would you have met these challenges/solved these challenges in a different way?

7. Now I would like to hear about the practicalities of being a participant in the study. By practicalities I mean things such as time consumption. How demanding or challenging has it been for you to take part in this study? On a scale from 0 to 10 where 0 is easy and not challenging and 10 is demanding or very challenging.
    a. What type of challenges have you encountered?
    b. Are there anything that could have made it easier? (Internal and external challenges)

8. What are your thoughts on changing use of sleeping pills today?

9. What do you think is needed now in order for you to change use of sleeping pills?

10. When you think back at the conversation you had with the doctor that day. Do you think others that are using sleeping pills could benefit from such a conversation?
    a. Is there anything you remember that were particularly important or interesting?
    b. Is there anything you think could be done differently or improved?
